# Higher expression of FOXOs correlates to better prognosis of bladder cancer

**DOI:** 10.18632/oncotarget.22029

**Published:** 2017-10-24

**Authors:** Ying Zhang, Linpei Jia, Ying Zhang, Wei Ji, Hai Li

**Affiliations:** ^1^ Department of Pathology, China-Japan Union Hospital of Jilin University, Changchun 130033, P.R. China; ^2^ Department of Nephrology, Xuanwu Hospital, Capital Medical University, Beijing 100000, P.R. China; ^3^ Department of Neurology, First Hospital of Jilin University, Changchun 130021, P.R. China; ^4^ Department of Vascular Surgery, Jilin Provincial People's Hospital, Changchun 130000, P.R. China; ^5^ Department of Urology, China-Japan Union Hospital of Jilin University, Changchun 130033, P.R. China

**Keywords:** bladder cancer, forkhead box class O, clinicopathological characteristics, prognosis

## Abstract

**Background:**

We aimed to explore the expression of forkhead box class O (FOXO) and relations between expressions of FOXOs and clinicopathological characteristics and prognosis of bladder cancer.

**Methods:**

We enrolled a cohort of 276 patients with bladder cancer in our study. Expressions of FOXOs in bladder cancer tissue and adjacent tissue were measured by quantitative real-time polymerase chain reaction (qRT-PCR) and immunohistochemistry (IHC). Correlations between expression of FOXOs and clinicopathological characteristics and prognosis were analyzed. The relationship between expression of FOXOs and survival time of patients with bladder cancer was analyzed by the Kaplan-Meier survival analysis and the Log-rank test; individual variables which may affect the prognosis of bladder cancer were detected by the Cox proportional hazard regression model.

**Results:**

Compared with bladder cancer tissue, a higher expression of FOXOs was detected in paracancerous tissue. We found significant associations between histological grade and the expressions of FOXOs, clinical stage and the expressions of FOXOs, and lymph node metastasis and the expressions of FOXOs (all *P* < 0.05). When used for diagnosing bladder cancer, the mRNA expression of FOXO1/3/4 produced cut off values of 1.475, 1.305, and 1.295, respectively, exhibiting relatively high specificity and sensitivity. The Kaplan-Meier curves indicated that patients with a higher expression of FOXOs tended to have a longer overall survival than those with lower expression. The Cox regression analysis revealed that lymph node metastasis, high clinical stage, and low expression of FOXOs were independent risk factors for bladder cancer prognosis.

**Conclusion:**

Our results indicate that the expression of FOXOs is closely correlated with clinicopathological characteristics and prognosis of bladder cancer.

## INTRODUCTION

Arising from the epithelial layer of urinary bladder, bladder cancer is one of the most common malignant cancers in the world and its incidence has gradually increased, listed as the sixth most frequent form of cancer in 2013 [1]. About 110,500 males and 70,000 females are diagnosed as new cases every year, causing about 38,200 and 17,000 deaths in the European Union and in the USA, respectively [2]. About 80% patients with bladder cancer are diagnosed as non-muscle-invasive bladder cancer when first diagnosed, and about 80% of these patients experience a recurrence after initial treatment within 5 years [3]. There are a few treatments for bladder cancer, for example, intravesical Bacillus Calmette-Guérin (BCG) is the main but controversial treatment because about 30%-40% of patients fail to respond to it [4]. Furthermore, neoadjuvant chemotherapy (NAC) also provides a significant survival benefit for the treatment of bladder cancer but it is limited because of chemotherapy-related toxicity and the delay of final local treatment [5].

Forkhead box class O (FOXO) is the subgroup O of forkhead box (FOX) transcription factors, which includes 4 members, FOXO1, FOXO3, FOXO4 and FOXO6 [6]. FOXOs play a crucial role in cell processes like cell proliferation, apoptosis, DNA repair and stress response [7]. FOXOs are closely related to the growth of cervical cancer cells through regulating the expression of certain proteins or genes [8]. FOXOs have highly conserved forkhead DNA-binding domain [9]. Combination of FOXO1 and p53 can provide relevant prognostic information on progression and recurrence of bladder cancer [10]. The transcription factor FOXO3 is an effective tumor suppressor; dysregulation of FOXO3 is associated with cancer initiation and progression [11]. FOXO3 and FOXO4 may play an important role in cell apoptosis and the regulation of cell cycle in fetal membrane rupture [12]. FOXO4 is known as a tumor suppressor protein, which is closely related to the metastasis of cholangiocarcinoma [13]. However, given the inadequate data about the relationship between FOXOs and bladder cancer, we explored the expression of FOXOs in bladder cancer tissue and paracancerous tissue, so as to elucidate the correlation between expression of FOXOs with clinicopathological features and prognosis of bladder cancer.

## RESULTS

### Demographic of patients

A cohort of 276 patients diagnosed with bladder cancer were finally enrolled, among whom 218 were males and 58 were females, with a median age of 57 (range: 21-76) years.

### Expressions of FOXOs in bladder cancer tissue and normal bladder tissue

**Figure 1:**
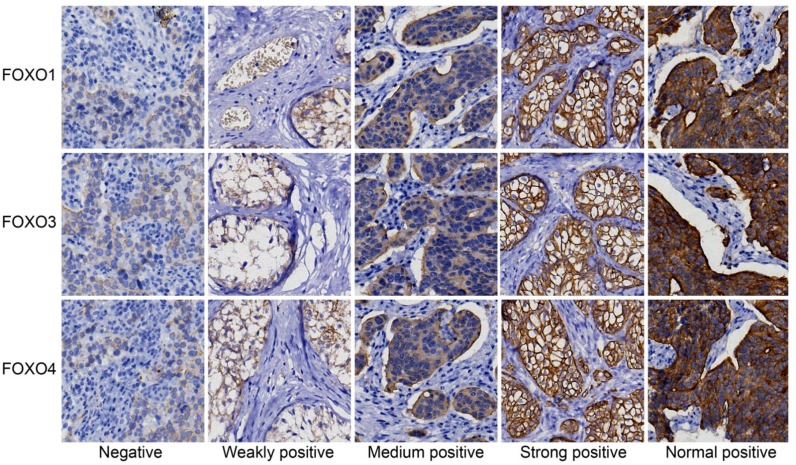
Expressions of FOXOs in cancer and paracancerous tissue, detected by immunohistochemistry The expressions of FOXO1, FOXO3 and FOXO4 are shown. The density of staining includes negative, weakly positive, moderately positive, strongly positive and normal positive. FOXO, forkhead box class O.

The expressions of FOXOs protein in bladder cancer tissue and paracancerous tissue were detected by IHC (Figure 1). The positive staining of FOXOs was mainly located in cytoplasm, and colored brown-yellow. Bladder cancer tissues exhibited more FOXOs-negative cells than paracancerous tissue. In the 276 tumor tissue samples, negative expression of FOXO1 was found in 150 cases, weakly positive in 34 cases, moderate level in 62 cases, and strongly positive in 30 cases; negative expression of FOXO3 was found in 134 cases, weakly positive in 44 cases, moderately positive in 36 cases, and strongly positive in 62 cases; negative expression of FOXO4 was found in 118 cases, weakly positive in 60 cases, moderately positive in 52 cases, and strongly positive in 46 cases.

### Association between protein expression of FOXOs and clinical features of bladder cancer

Significant differences were found in comparisons of the expressions of FOXOs with histological grade, clinical stage and lymph node metastasis (all *P* < 0.05). However, no significant difference was found in comparisons of low expression of FOXOs with patients’ gender and age, as well as tumor size and number (all *P* > 0.05) (Table 1).

**Table 1:** Cox regression model of multiple variables for bladder cancer prognosis

	Univariate analysis				Multivariate analysis			
Factor	Exp.(B)	95% CI		*P*	Exp.(B)	95% CI		*P*
		Lower limit	Upper limit			Lower limit	Upper limit	
Gender	0.876	0.571	1.344	0.545				
Age	0.843	0.596	1.194	0.337				
Tumor size	1.178	0.840	1.654	0.342				
Tumor number	1.369	0.967	1.939	0.077				
Histological grade	1.348	0.928	1.958	0.116	1.375	0.911	2.074	0.129
Clinical stage	2.814	1.918	4.128	<0.001	1.861	1.217	2.844	0.004
Lymph node metastasis	2.914	2.067	4.109	<0.001	1.866	1.284	2.712	0.001
Expression of FOXO1	0.085	0.050	0.144	<0.001	0.333	0.170	0.654	0.001
Expression of FOXO3	0.135	0.088	0.205	<0.001	0.434	0.261	0.724	0.001
Expression of FOXO4	0.113	0.076	0.168	<0.001	0.314	0.196	0.505	<0.001

Cox, Cox's proportional hazards regression model; FOXO, forkhead box class O.

### Expression of FOXOs mRNA in bladder cancer tissue and paracancerous tissue

**Figure 2:**
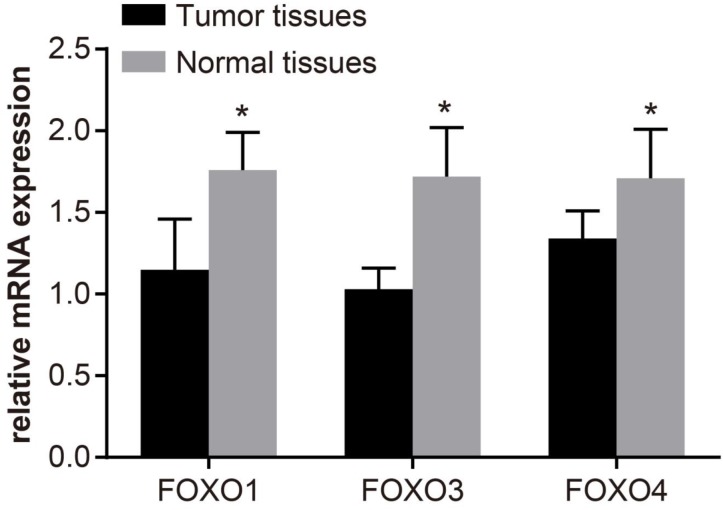
The mRNA expression levels of FOXOs in bladder cancer tissue and paracancerous tissue, determined by quantitative real-time polymerase chain reaction (qRT-PCR) The q-PCR assay was used to test the mRNA expression of FOXO1, FOXO3 and FOXO4 in both normal and tumor tissues, and the results showed that FOXO1, FOXO3 and FOXO4 in tumor tissues had a lower expression than normal tissues; ^*^, *P* < 0.05 compared with paracancerous; FOXO, forkhead box class O.

As shown in Figure 2, FOXOs were over-expressed in paracancerous tissue, and their expressions are significantly higher than in bladder cancer tissue (*P* < 0.05). Compared with low-grade patients, the expressions of FOXOs in high-grade patients were significantly decreased (*P* < 0.05). Importantly, the FOXOs expressions in patients with superficial bladder cancer were higher than patients with invasive bladder cancer (*P* < 0.05). Moreover, the FOXOs expressions in patients without lymph node metastasis were significantly higher than patients with lymph node metastasis (*P* < 0.05).

### Diagnostic value of mRNA expression of FOXOs in bladder cancer

**Figure 3:**
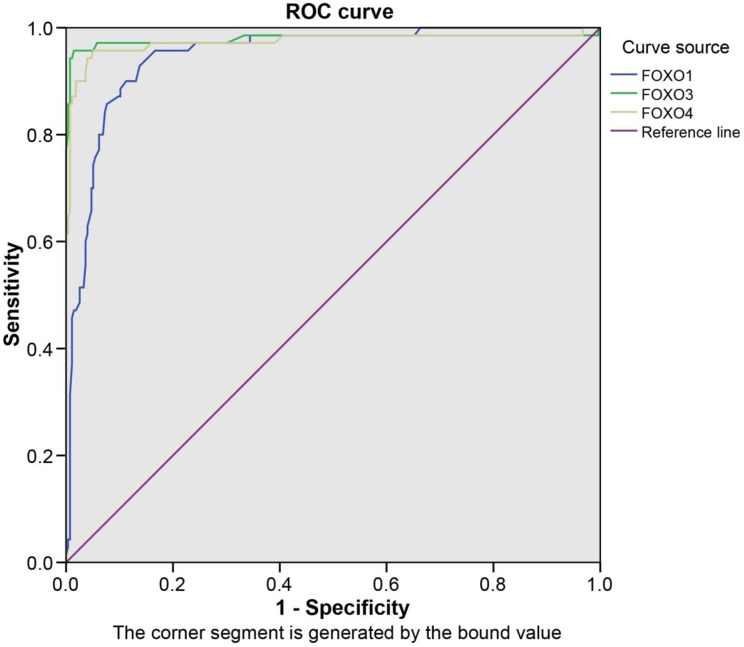
The ROC curves of FOXOs in patients with bladder cancer The ROC curve was drawn using sensitivity as the ordinate and specificity as the abscissa, and the area under curve was shown. The mRNA expressions of FOXO1/3/4, as the value of cutoff for diagnosing, were 1.475, 1.305, 1.295, respectively, by which we could confirm a patient with bladder cancer. ROC, receiver operating characteristic; FOXO, forkhead box class O.

The sensitivity and the specificity of mRNA expression of FOXOs in bladder cancer were shown in Table 2. The ROC curve was drawn using sensitivity as the ordinate and specificity as the abscissa, and the area under the curve was shown in Figure 3 (*P* < 0.05). The mRNA expressions of FOXO1/3/4, as the cutoff values for diagnosing, were 1.475, 1.305, 1.295, respectively, and there was the highest sensitivity and specificity when detected by qRT-PCR. When the mRNA expressions of FOXO1/3/4 were less than 1.475, 1.305 and 1.295, respectively, we could primarily diagnose the patient with bladder cancer.

**Table 2:** Correlation of FOXOs expressions of and clinical features

Group	n = 276	FOXO1 expression			FOXO3 expression			FOXO4 expression		
		Negative	Positive	*P*	Negative	Positive	*P*	Negative	Positive	*P*
Age (year)										
<60	160	80	80	0.089	72	88	0.166	76	84	0.061
≥60	116	70	46		62	54		42	74	
Gender										
Male	218	120	98	0.652	104	114	0.586	92	126	0.719
Female	58	30	28		30	28		26	32	
Tumor size										
< 3cm	146	80	66	0.875	76	70	0.217	64	82	0.7
≥ 3cm	130	70	60		58	72		54	76	
Tumor number										
Single	186	102	84	0.814	92	94	0.663	80	106	0.901
Multiple	90	48	42		42	48		38	52	
Histological grade										
Low grade	90	38	52	0.005	32	58	0.003	27	63	0.004
High grade	186	112	74		102	84		91	95	
Clinical stage										
T_A_+T_1_	124	48	76	<0.001	46	78	0.001	42	82	0.007
T_2_~T_4_	152	102	50		88	64		76	76	
Lymph node metastasis										
No	196	90	106	0.002	84	112	0.003	66	130	< 0.001
Yes	80	60	20		50	30		52	28	

FOXO, FOXO, forkhead box class O.

### Association between the expression of FOXOs and the prognosis of bladder cancer

**Figure 4:**
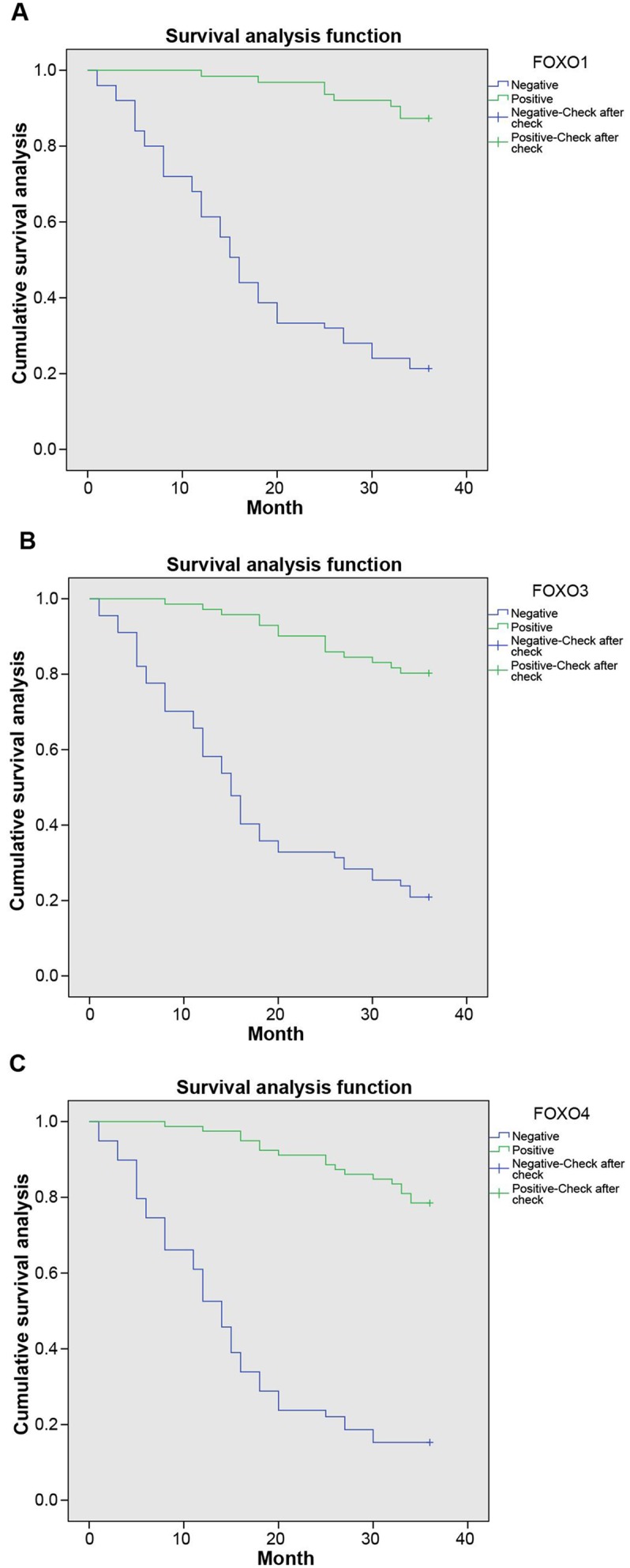
The Kaplan-Meier curve of associations between expression of FOXOs and survival time of patients with bladder cancer The results of survival analysis revealed that patients with positive expressions of FOXO1 had higher survival than patients with negative expressions **(A)**. More importantly, similar results can be seen in FOXO3 and FOXO4 **(B and C)**, suggesting FOXO1, FOXO3 and FOXO4 may play a negative role to promote the progression of bladder cancer. A, FOXO1; B, FOXO3; C, FOXO4; FOXO, forkhead box class O.

The association between expression of FOXOs and survival time of patients with bladder cancer was analyzed by the Kaplan-Meier method. Results indicated that the survival curve of the group with low expression of FOXOs was below the one with high expression and the overall survival of the low-expression FOXOs was shorter than the high-expression FOXOs (*P* <0.05, Figure 4).

### Evaluation of risk factors for prognosis of bladder cancer

The Cox regression model was used to analyze individual variables which may affect the prognosis of bladder cancer. Except for the expression of FOXOs, other variables included gender, age, tumor size, number, grade, stage and lymph node metastasis. Univariate analysis indicated that the overall survival was related to histological grade, clinical stage, lymph node metastasis and expression of FOXOs. Further multivariate analysis was used to analyze these statistically significant variables; the results indicated that lymph node metastasis, high clinical stage, and low expression of FOXOs were the risk factors which affect the prognosis of bladder cancer (both *P* < 0.05) (Table 3).

**Table 3:** ROC curve of FOXOs mRNA expressions

Variable	Sensitivity (%)	Specificity (%)	AUC	*P*	cutoff
FOXO1	92.9	86.2	0.948	< 0.001	1.475
FOXO3	95.7	98.6	0.979	< 0.001	1.305
FOXO4	95.7	94.9	0.974	< 0.001	1.295

ROC, receiver operating characteristic; AUC, area under the curve; FOXO, FOXO, forkhead box class O.

## DISCUSSION

In the present study, we explored the correlation between the expression of FOXOs and prognostic factors in bladder cancer. We found that expression of FOXOs increased with higher histological grade and clinical stage, while significantly decreased in patients with lymph node metastasis. Low expression of FOXOs could be regarded as an individual risk factor to predict bladder cancer prognosis.

Bladder cancer is a heterogeneous disease. The disease is prone to relapse in about 70% of patients although not life threatening, and muscle-invasive with high risk of distant metastases and death in the other 30% of patients” [14]. The survival rate after surgery for 5 years was about 25%-80%, which is even worse in patients with lymph node metastasis [15]. Therefore, understanding the biological mechanism underlying bladder cancer may help find new biomarkers for establishing effective strategy in prevention, early diagnosis and treatment of bladder cancer. A former study found that FOXOs are involved in many processes such as tumor suppression and cell apoptosis [16]. In our study, compared with bladder cancer tissue, overexpression of FOXOs was found in paracancerous tissue. The FOXOs family including FKHR (FOXO1), FKHRL1 (FOXO3a), and AFX (FOXO4), are characterized by a conserved DNA-binding domain [17]. In animal experiments, mice without somatic cells of FOXO1, FOXO3a, and FOXO4 led to the progression of hemangiomas and thymic lymphomas, which proved the possible function of FOXO to serve as the redundant inhibitors of tumor growth [18]. The low-expressed FOXOs play a crucial role in tumor progression via down-regulation of target genes related to stress resistance, cell cycle arrest and cell apoptosis [6]. In bladder cancer, down-regulation of FOXO1 was closely connected with worse results like high risk of recurrence, especially in high-grade tumors [10]. A previous study suggested that the activation of FOXO3a was related to lymph node metastasis and poor prognosis in patients with breast cancer [19].

Furthermore, we found that low expression of FOXOs is related to higher histological grade, clinical stage, and lymph node metastasis. FOXO3a can regulate motility of urothelial cancer by down-regulating Twist2 and YB-1, and up-regulating E-cadherin [20]. Yu et al found that Nkx 2.8, as one of the tumor suppressor, inhibited bladder cancer proliferation through up-regulating the expression of FOXO3a [21]. The expression of inactivated form of phosphorylated FOXO1 was inversely related to lymph node metastasis and positively associated with a longer survival time in patients with early-stage gastric cancer [22]. The expression of FOXO1 was enhanced in superficial bladder cancer when compared with invasive cancer, and compared with the high-grade group (grade 3), expression of FOXO1 was higher in low-grade bladder cancer (grade 1-2) [23]. Moreover, the expression of nuclear FOXO3a was related to a low histological grade, which supported the inhibitory effect of FOXO3a [24]. Additionally, patients with high expression of FOXOs could survive longer than those with low expressions of FOXOs. FOXOs can facilitate DNA repair through regulating defined transcriptional programs and executing transcription-independent functions, contributing to genomic stability and further longevity and tumor-free survival [25]. For these reasons and former studies, we may infer that in cancer cells, FOXOs could serve as a suppressor of tumor progression. Understanding the regulation of FOXOs may yield alternative methods for the diagnosis and treatments of bladder cancer. However, the detailed mechanism of FOXOs still needs further investigation.

In conclusion, expression of FOXOs was significantly decreased with higher histological grade and clinical stage and lymph node metastasis, and low expression of FOXOs was found as one of the individual risk factors of bladder cancer prognosis. Thus, FOXOs may serve as a new biomarker for diagnosing bladder cancer. However, due to the complicated individual risk factors of bladder cancer, adequate experimental data to elucidate the specific mechanism of FOXOs in bladder cancer are still lacking, and further studies are needed.

## MATERIALS AND METHODS

### Study subjects and grouping

Between June 2009 and June 2013, a total of 276 patients diagnosed with bladder cancer were enrolled into the study. All the patients were treated with surgical treatment (transurethral resection or radical cystectomy for bladder cancer). Besides, 35 samples of the corresponding non-cancerous mucosa, which was 2 cm away from the tumor were collected for the study. Inclusion criteria: first, patients were diagnosed with transitional cell carcinoma (TCC) by pathologic examination; second, complete clinical data, pathological data and follow-up data were available; third, patients did not receive any chemotherapy or radiotherapy, including intravesical instillation. The follow-up time was 3 years, with the first 2 years being followed up every 3 months and every 6 months in the last year. Collected clinical data of patients included age, gender, survival time, tumor size and number, tumor grade, tumor stage, lymph node metastasis, etc. This study was approved by the ethics committee of China-Japan Union Hospital of Jilin University, and all the patients signed the informed consent.

### Immunohistochemistry (IHC)

Bladder cancer tissue and normal bladder tissue were fixed, dehydrated, embedded in paraffin, and sliced up (the thickness of slice was 4 μm). The sections were blocked with endogenous peroxidase with 1% H_2_0_2_, put into antigen repair solution, heated to 97°C for 15 min and cooled naturally. The samples were incubated with normal goat serum (Diagnostics Scotland, Edinburgh, UK) blocking solution at room temperature for 20 min, with the excess solution threw away. Then the sections were added with FOXO1 antibody (Abcam Company, Cambridge, MA, USA), FOXO3 antibody (Bioss Company, Beijing, China), FOXO4 antibody (Abcam Company, Cambridge, MA, USA) successively and incubated overnight at 4°C. Next, biotinylated secondary antibody (Zhongshan Golden Bridge Company, Beijing, China) was added and the tissues were incubated at 37°C for 40 min, followed by developing by diaminobenzidine (DAB) (Zhongshan Golden Bridge Company, Beijing, China). Positive sections were regarded as positive controls, and phosphate buffer saline (PBS) was used in place of the first antibody as negative controls. Multifunctional Real Color Image Analysis System (Media Cybernetics Company, Maryland, USA) was used for analysis. Five high power fields were selected randomly in each section, and the expression was calculated based on positive staining rate and color depth: the cells were scored according to the color depth: 0 is colorless, 1 is pale yellow, 2 is brown, and 4 is dark brown. Cells were also scored based on the percentage of staining cells in counting cells: 0 is no positive cells; 1, 2 and 3 indicate < 10%, 11%-50%, and > 50% positive staining cells, respectively. By multiplying these two together, 0 is negative, 1-2 indicate weakly positive, 3-4 indicate moderately positive, 5-12 indicate strongly positive. Color depth and relevant score of each sample were evaluated independently by two pathologists in a double-blind way. The average number was used as the protein expression (expression intensity) of the corresponding sample.

### Quantitative real-time polymerase chain reaction (qRT-PCR)

The total RNAs from the cancer tissue and normal bladder tissue were extracted by the Trizol total RNA extraction kit (Aidlab Company, Beijing, China). The RNA samples (5 μL) were collected and diluted 20 times by ultra-pure RNase-free water. The absorbance at 260 nm and 280 nm on ultraviolet spectrophotometer was observed and the concentration and the purity of RNA were measured. The ratio of OD260/OD280 between 1.7 and 2.1 indicates a relatively high RNA purity to meet the needs of subsequent experiments. Complementary DNA (cDNA) was synthesized by reverse transcription reaction, and qRT-PCR experiment was conducted using ABI7500 quantitative PCR instrument (Applied Biosystem, Grand Island, NY, USA), the reaction conditions were as follows: pre-denaturation at 95°C for 10 min, 45 PCR cycles (95°C for 15 s, 60°C for 1 min). The primer sequences are shown in Table 4. β-actin was regarded as internal reference, and 2^-ΔΔCt^ presented the ratio of gene expression between the observation group and the control group. The formula was ΔΔCT = ΔCt _observation group_ - ΔCt _control group_, ΔCt = Ct _target gene -_ Ct _β-actin_. The threshold cycle (CT) was the amplification cycles when real-time fluorescence intensity reached the set threshold, and at this point, the growth rate was logarithmic.

**Table 4:** RT-PCR Primer sequences for qRT-PCR

Gene	Primer sequence
FOXO1	5’-AACCTGGCATTACAGTTGGCC -3’
	5’-AAATGCAGGAGGCATGACTACGT-3’
FOXO3	5’-AAGCCAGCTACCTTCTCTTCCA -3’
	5’- GTGGCTAAGTGAGTCCGAAGTGA-3’
FOXO4	5’-CCCGACCAGAGATCGCTAAC-3’
	5’-TCCGTGTGTACCTTTTCCCC-3’
β-actin	5’- CTCCTTAATGTCACGCAGGATTTC -3’
	5’- GTGGGGCGCCCCAGGCACCA-3’

qRT-PCR, quantitative real-time polymerase chain reaction; FOXO, forkhead box class O.

### Statistics

The Statistical Program for Social Sciences (SPSS) 19.0 software (SPSS, IBM, West Grove, PA, USA) was used for data analysis. Measurement data were expressed as mean ± standard deviation, and enumeration data were expressed as rate or percentage. Comparisons of measurement data and enumeration data were statistically analyzed with the Student-t test and the χ^2^ test, respectively. Diagnostic efficiency was analyzed by receiver operating characteristic (ROC) curve. The relationship between the survival time of patients after surgery and expression of FOXOs was analyzed by the Kaplan-Meier method. The individual variables which affect the survival of patients with bladder cancer were evaluated by Cox proportional hazard regression model. The statistical significance of univariate and multivariate Cox regression model was confirmed by Wald's test. All tests were two-tailed, with the level of significance set to *P* < 0.05.

## Abbreviations

FOXO: forkhead box class O
IHC: immunohistochemistry
NAC: neoadjuvant chemotherapy
qRT-PCR: quantitative real-time polymerase chain reaction
